# New Therapeutic Strategy for Overcoming Multidrug Resistance in Cancer Cells with Pyrazolo[3,4-*d*]pyrimidine Tyrosine Kinase Inhibitors

**DOI:** 10.3390/cancers13215308

**Published:** 2021-10-22

**Authors:** Ana Podolski-Renić, Jelena Dinić, Tijana Stanković, Ivanka Tsakovska, Ilza Pajeva, Tiziano Tuccinardi, Lorenzo Botta, Silvia Schenone, Milica Pešić

**Affiliations:** 1 Department of Neurobiology, Institute for Biological Research “Siniša Stanković”—National Institute of Republic of Serbia, University of Belgrade, Despota Stefana 142, 11060 Belgrade, Serbia; ana.podolski@ibiss.bg.ac.rs (A.P.-R.); jelena.dinic@ibiss.bg.ac.rs (J.D.); tijana.andjelkovic@ibiss.bg.ac.rs (T.S.); 2 QSAR & Molecular Modelling Department, Institute of Biophysics & Biomedical Engineering, Bulgarian Academy of Sciences, Acad. G. Bonchev Str., Bl. 105, 1113 Sofia, Bulgaria; itsakovska@biomed.bas.bg (I.T.); pajeva@biomed.bas.bg (I.P.); 3 Department of Pharmacy, University of Pisa, Via Bonanno 6, 56126 Pisa, Italy; tiziano.tuccinardi@unipi.it; 4 Department of Ecological and Biological Sciences, University of Tuscia, Via S.C. De Lellis snc 44, 01100 Viterbo, Italy; lorenzo.botta@unitus.it; 5 Lead Discovery Siena s.r.l. (LDS), Via Vittorio Alfieri, 31, Castelnuovo Berardenga, 53019 Siena, Italy; 6 Department of Pharmacy, University of Genova, Viale Benedetto XV 3, 16132 Genova, Italy; schenone@difar.unige.it

**Keywords:** cancer, multidrug resistance, P-glycoprotein inhibitors, Src family tyrosine kinase inhibitors

## Abstract

**Simple Summary:**

P-glycoprotein (P-gp) is an ATP-binding cassette transporter whose overexpression in cancer cells is one of the main causes of multidrug resistance (MDR). Tyrosine kinase inhibitors (TKIs) have been reported to interact with ABC transporters and in some cases, increase the susceptibility of cancer cells to chemotherapy. We investigated the potential of novel TKI pyrazolo[3,4-*d*] pyrimidines and their prodrugs to inhibit P-gp in two MDR cancer cell lines with P-gp overexpression. The tested compounds were able to suppress P-gp by inhibiting its ATPase activity. Interestingly, prodrugs displayed a stronger potential to modulate P-gp and showed higher interaction energies in the docking simulations compared to their parent drugs. Furthermore, prodrugs showed significant potential to inhibit P-gp activity even in prolonged treatment and therefore to enhance the efficacy of doxorubicin and paclitaxel in MDR cancer cells. All of these characteristics imply that the new TKIs could be considered a valuable strategy for combating resistant cancers, especially in combination with other chemotherapeutics.

**Abstract:**

Tyrosine kinase inhibitors (TKIs) often interact with the multidrug resistant (MDR) phenotype of cancer cells. In some cases, TKIs increase the susceptibility of MDR cancer cells to chemotherapy. As the overexpression of membrane transporter P-glycoprotein (P-gp) is the most common alteration in MDR cancer cells, we investigated the effects of TKI pyrazolo[3,4-*d*]pyrimidines on P-gp inhibition in two cellular models comprising sensitive and corresponding MDR cancer cells (human non-small cell lung carcinoma and colorectal adenocarcinoma). Tested TKIs showed collateral sensitivity by inducing stronger inhibition of MDR cancer cell line viability. Moreover, TKIs directly interacted with P-gp and inhibited its ATPase activity. Their potential P-gp binding site was proposed by molecular docking simulations. TKIs reversed resistance to doxorubicin and paclitaxel in a concentration-dependent manner. The expression studies excluded the indirect effect of TKIs on P-gp through regulation of its expression. A kinetics study showed that TKIs decreased P-gp activity and this effect was sustained for seven days in both MDR models. Therefore, pyrazolo[3,4-*d*]pyrimidines with potential for reversing P-gp-mediated MDR even in prolonged treatments can be considered a new therapeutic strategy for overcoming cancer MDR.

## 1. Introduction

One of the major limitations for successful cancer treatment is the development of multidrug resistance (MDR). The concept of MDR indicates that cancer cells become resistant not only to applied drugs but also to a broad array of compounds with unrelated structures and modes of action [1]. MDR is commonly associated with the overexpression of ATP-binding cassette (ABC) transporters in the cancer cell membrane. ABC transporters utilize the energy from ATP hydrolysis to extrude drugs against their concentration gradients [2]. Members of the ABC transporter family that have been clearly correlated with MDR are P-glycoprotein (P-gp/ABCB1), multidrug resistance protein 1 (MRP1/ABCC1), and breast cancer resistance protein (BCRP/BCG2) [1].

P-gp is a 170 kD glycoprotein encoded by the *ABCB1* gene. Besides expression on cancer cell membranes, P-gp is expressed in normal cells such as excretory epithelial cells of the liver, gastrointestinal tract, and kidney and in endothelial cells of physiological barriers such as the blood–brain barrier (BBB) [3]. P-gp transports a broad range of xenobiotic compounds, including clinically important chemotherapeutics and tyrosine kinase inhibitors (TKIs) [1,4]. Functional P-gp contains two homologous transmembrane domains (TMDs) and two homologous intracellular nucleotide-binding domains (NBDs) or ATP-binding cassettes [5]. The two TMDs are composed of twelve transmembrane α-helices that form a huge, central pathway for drug-binding and efflux. The huge and highly flexible transmembrane drug-binding pocket of P-gp enables simultaneous binding of different compounds as well as binding of one compound to several distinct sites [4]. Each NBD contains ATPase sites, which directly mediate the binding and hydrolysis of ATP [5]. NBDs and TMDs are connected through a linker region that plays a significant role in coupling ATP hydrolysis to drug export [6]. As a transporter with high promiscuity, P-gp is an attractive drug target in cancer cells with P-gp overexpression. P-gp activity could be suppressed when ATP hydrolysis is highly stimulated due to overloading by its substrate or when ATP hydrolysis is suppressed [7].

Small-molecule TKIs are a new generation of anticancer drugs [8]. These hydrophobic molecules easily penetrate into cells and inhibit both membrane-bound and cytoplasmic tyrosine kinases (TKs), which regulate multiple signaling pathways involved in cell survival, proliferation, and differentiation [9]. Since the deregulation of TK activity is crucial for cancer development and progression, TKs have become one of the most important drug targets in the 21st century. In 2020, eight TKIs were approved by the FDA [10]. Depending on the concentration, applied TKIs may interact with ABC transporters either as their substrates or inhibitors. At lower concentrations, most TKIs are substrates for ABC transporters. However, at higher concentrations TKIs act as competitive inhibitors of ABC transporters by overloading the transporter’s drug-binding pocket [11]. Alternatively, some TKIs act as true inhibitors of ABC transporters by binding to their ATP-binding site, thus affecting the transporter’s ATPase activity [12]. Moreover, TKIs may also change the expression level of ABC transporters [11].

A novel series of TKIs based on the pyrazolo[3,4-*d*]pyrimidine scaffold have been reported. These ATP-competitive TKIs act against the Src family tyrosine kinases (SFK) [13,14]. A number of SFK inhibitors exhibited significant anticancer activity in vitro against different cancer cell lines [15,16,17] as well as in vivo [18,19,20]. We previously reported that SFK inhibitors Si306 and its prodrug pro-Si306 act as dual targeting molecules showing the ability to inhibit both SFK and P-gp in glioblastoma cells [21].

**Figure 1:**
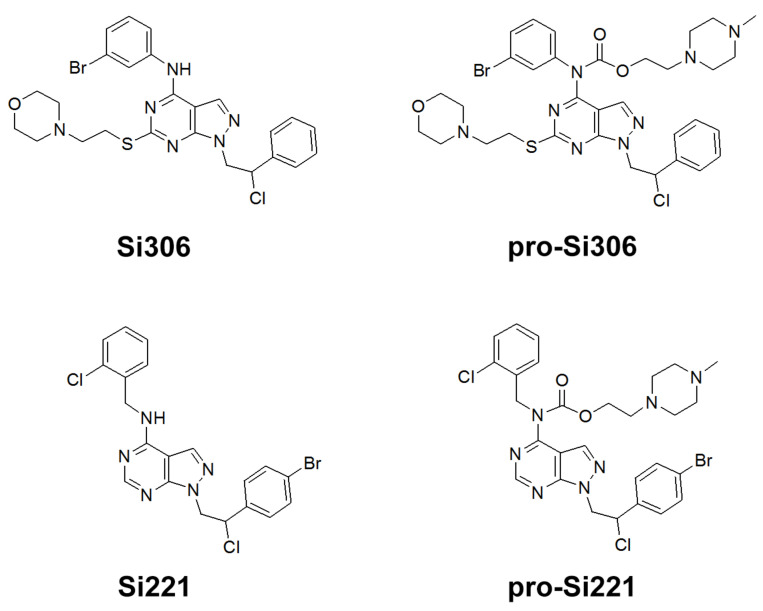
Chemical structures of Si306, pro-Si306, Si221, and pro-Si221.

Our previous study inspired us to evaluate whether Si306 and its prodrug pro-Si306 as well as two other SFK inhibitors, Si221 and its prodrug pro-Si221 (Figure 1), interact with P-gp in different tumor types. Therefore, this study was conducted on two MDR models composed of P-gp overexpressing cancer cells (non-small cell lung carcinoma NCI-H460/R and colorectal carcinoma DLD1-TxR) [22]. These MDR models were established from NCI-H460 and DLD1 by continuous selective pressure of doxorubicin (DOX) and paclitaxel (PTX), respectively. We investigated concentration- and time-dependent effects of SFK inhibitors on P-gp-mediated efflux, their ability to modulate MDR, as well as their effect on P-gp expression. Docking studies were conducted to identify the potential P-gp binding site of SFK inhibitors.

## 2. Results

### 2.1. SFK Inhibitors Decrease Cell Viability

The effects of the novel SFK inhibitors on the cell viability of two human sensitive/MDR cancer cell line pairs (non-small cell lung carcinoma and colorectal carcinoma) were assessed by MTT assay. The results are compared to the well-known SFK inhibitor dasatinib and are summarized in Table 1. Si306, pro-Si306, and pro-Si221 as well as dasatinib showed considerable efficacy with IC_50_ values less than 10 µM. Only Si221 was less potent than dasatinib. Collateral sensitivity to SFK inhibitors was observed in colorectal carcinoma cells owing to the fact that MDR DLD1-TxR cells were more sensitive to all analyzed SFK inhibitors compared to DLD1 cells. Importantly, Si306 and pro-Si306 showed significant selectivity toward MDR NCI-H460/R cells with more than 2-fold lower IC_50_ values than those obtained in the corresponding sensitive NCI-H460 cells.

### 2.2. SFK Inhibitors Modify P-gp Activity

**Figure 2:**
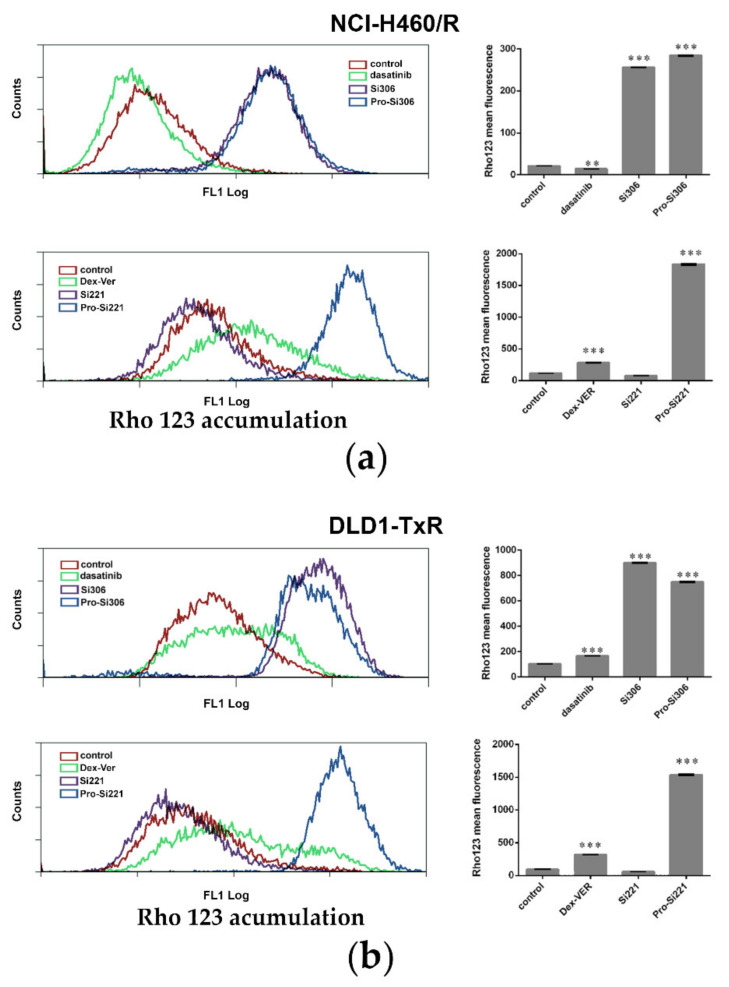
SFK inhibitors suppress P-gp activity. Flow-cytometric profile and the mean fluorescence intensity of Rho123 accumulation assessed after 30-min treatment with 10 µM Si306, pro-Si306, Si221, and pro-Si221 in (**a**) NCI-H460/R and (**b**) DLD1-TxR cell lines. Dasatinib and Dex-VER are included as reference compounds. Three independent experiments were performed, and a minimum of 10,000 events were collected for each experimental sample. The fluorescence intensity of Rho123 accumulation is presented as the mean ± SEM. Statistically significant differences between untreated control and treated samples: *p* < 0.01 (**), *p* < 0.001 (***).

To investigate the effect of SFK inhibitors on P-gp activity in MDR cancer cells, intracellular accumulation of P-gp substrate rhodamine 123 (Rho 123) was analyzed by flow cytometry after 30-min treatment (Figure 2). The accumulation of Rho123 was compared with dasatinib and Dex-verapamil (Dex-VER), a well-known competitive P-gp inhibitor. All compounds were applied at 10 µM. According to a marked increase in Rho 123 intracellular accumulation, the inhibition of P-gp achieved by Si306, its prodrug pro-Si306, and pro-Si221 was considerably more potent than that observed with Dex-VER in NCI-H460/R cells (Figure 2a) and DLD1-TxR cells (Figure 2b). Si221 did not modulate the activity of P-gp (Figure 2), while dasatinib showed the similar potential as Dex-Ver only in DLD1-TxR cells (Figure 2b).

**Figure 3:**
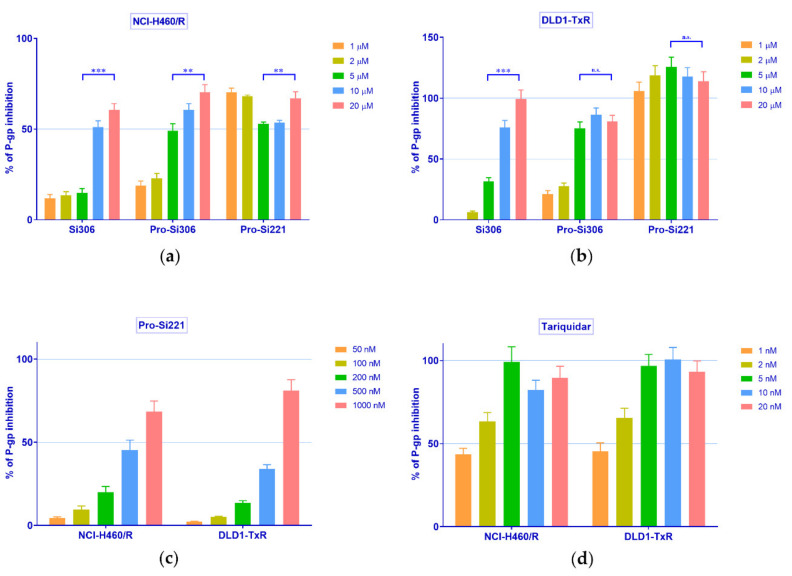
Concentration-dependent inhibition of P-gp activity. Rho 123 accumulation in (**a**) NCI-H460/R and (**b**) DLD1-TxR cell lines was assessed after the application of increasing concentrations of Si306, pro-Si306, and pro-Si221 (1, 2, 5, 10, 20 µM). The lower range of concentrations for (**c**) pro-Si221 (0.05, 0.1, 0.2, 0.5, 1 μM) and (**d**) TQ (1, 2, 5, 10, 20 nM) in NCI-H460/R and DLD1-TxR cells. The percentage of P-gp inhibition is expressed as an increase in Rho 123 accumulation; 100% of P-gp inhibition is considered when the level of Rho123 accumulation achieves the level of Rho123 accumulation in sensitive cells. Three independent experiments were performed (a minimum of 10,000 events were collected for each experimental sample). A statistically significant difference between 5 µM and 20 µM treatments with Si306, pro-Si306, and pro-Si221 is presented in (**a**) and (**b**): *p* < 0.01 (**), *p* < 0.001 (***), not significant (n.s.).

Therefore, the concentration-dependent inhibition of P-gp activity was further studied with Si306, pro-Si306, and pro-Si221 in NCI-H460/R and DLD1-TxR cells. To that end, the accumulation of Rho 123 was examined in MDR cancer cells after treatment with increasing concentrations of SFK inhibitors and compared with the effects of the known P-gp inhibitor tariquidar (TQ) (Figure 3). In a given range of concentrations (1–20 µM), Si306 and pro-Si306 inhibited P-gp function in a concentration-dependent manner (Figure 3a,b). Pro-Si221 achieved 100% of P-gp inhibition already after the application of 1 µM. Therefore, the lower concentration range of pro-Si221 was applied and the concentration-dependent effect of P-gp inhibition was achieved in both MDR cancer cell lines within the range of 50–1000 nM (Figure 3c). The TQ effect was studied within the range 1–20 nM (Figure 3d).

Upon the statistical analysis of the results presented in Figure 3, the concentration of SFK inhibitors and TQ necessary to inhibit P-gp activity by 50% (IC_50_ of P-gp inhibition) was determined. The IC_50_ values of P-gp inhibition for Si306 were 13.3 µM and 6.4 µM in NCI-H460/R and DLD1-TxR cells, respectively (Table 2). Significantly lower IC_50_ values of P-gp inhibition were observed for pro-Si306, 6.2 µM and 3 µM, respectively (Table 2), while pro-Si221 showed the highest potential to inhibit P-gp with IC_50_ values around 0.6 µM in both MDR cancer cell lines (Table 2).

**Figure 4:**
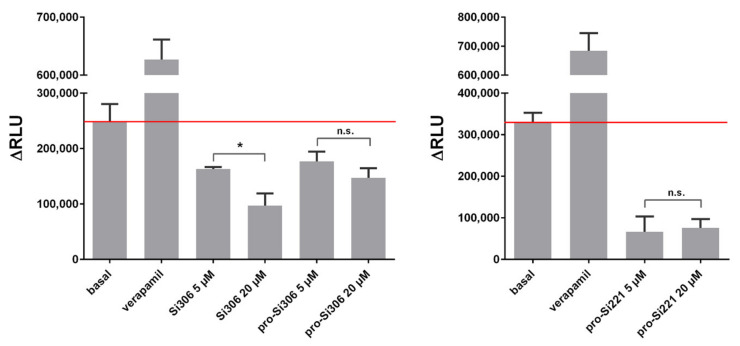
SFK inhibitors affect the ATPase activity of P-gp. The samples were treated with 5 µM and 20 µM Si306, pro-Si306, and pro-Si221. Verapamil was used as a positive control. The *y*-axis represents the difference in relative light units (ΔRLU) between Na3VO4-treated samples and samples treated with the tested compounds. ΔRLU basal is the difference in the luminescent signal between Na3VO4-treated and untreated samples. The results are expressed as the mean ± SEM. Statistically significant differences between 5 µM and 20 µM treatments with Si306, pro-Si306, and pro-Si221: *p* < 0.05 (*), not significant (n.s.).

Further, the ability of Si306, pro-Si306, and pro-Si221 to inhibit the ATPase activity of P-gp was evaluated. The P-gp substrate and competitive inhibitor - verapamil was used as a positive control. The results are summarized in Figure 4. All three SFK inhibitors showed potential to inhibit the ATPase activity of P-gp, opposite to verapamil, which stimulated the ATPase activity. The inhibition obtained with 20 µM Si306 was significantly stronger than that obtained with 5 µM Si306, which is in line with the results presented in Figure 3 a,b. The effects of two concentrations (5 µM and 20 µM) were similar for pro-Si306 and pro-Si221, which is also in line with the results presented in Figure 3 a,b. The highest inhibitory effect on ATPase activity was exerted by pro-Si221 (Figure 4).

### 2.3. The Effect of SFK Inhibitors on P-gp Expression

**Figure 5:**
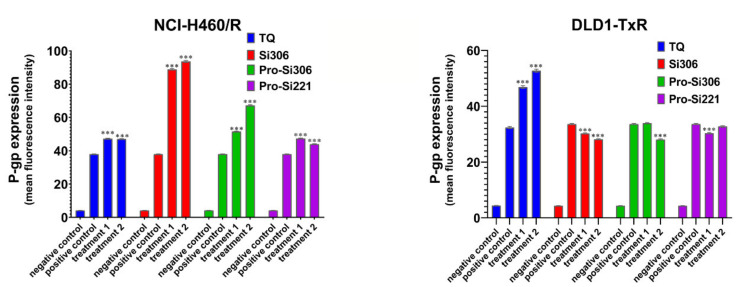
The effect of SFK inhibitors on P-gp expression at the protein level. Mean fluorescence intensity of primary P-gp antibody in NCI-H460/R and DLD1-TxR cells assessed after 48-h treatment with 5 µM and 10 µM (treatment 1 and treatment 2) for Si306 and pro-Si306 as well as 1 µM and 2 µM (treatment 1 and treatment 2) for pro-Si221. TQ is included as a reference compound with 5 nM for treatment 1 and 10 nM for treatment 2. The negative control is the auto-fluorescence of unstained untreated cells, whereas the positive control represents the fluorescence of anti-P-gp-labeled untreated cells. The results are expressed as the mean ± SEM. Statistically significant differences between the untreated control and treated samples calculated from three independent experiments: *p* < 0.001 (***).

To determine the influence of SFK inhibitors on P-gp expression in NCI-H460/R and DLD1-TxR cells, the expression of the protein product was analyzed by flow-cytometry after 48-h treatment with Si306, pro-Si306, pro-Si221, and TQ (Figure 5). Si306 significantly increased P-gp expression in NCI-H460/R cells, while a less pronounced effect on the increase in P-gp expression was observed after treatment with pro-Si306 and pro-Si221 (Figure 5). SFK inhibitors showed a tendency to decrease the P-gp expression in DLD1-TxR cells (Figure 5), while TQ increased the expression of P-gp in both MDR cancer cell lines (Figure 5).

### 2.4. Docking Studies

Keeping in mind that the successful development of P-g inhibitors requires an understanding of their interactions at a molecular level, we performed the docking of compounds Si221 and Si306 and their prodrugs, pro-Si221 and pro-Si306, into the binding cavity of P-gp.

Before docking, the compounds were analyzed for their ability to appear in ionized forms at physiological pH = 7.4, as described in Materials and Methods. The results are summarized in Table 3.

We further performed the docking of both neutral and protonated forms (where available) of the studied compounds into the binding cavity of the protein structure. For comparison, TQ was also docked as a well-known P-gp inhibitor. Table 4 summarizes the obtained docking scores S. The S-values for the 1st and 10th docking poses kept during the simulation are reported, and the 1st one corresponds to the best score calculated. The results allow outlining the S-trend based on the compounds’ neutral forms only due to the fact that Si221 was not protonated and the calculated prodrugs pro-Si221 and pro-Si306 appeared with different levels of protonation at physiological pH (Table 3). In addition, the neutral forms were more probable considering the predominant hydrophobic environment in the protein cavity.

In general, the ranking of the neutral forms based on the S values of the compounds is the following: pro-Si221 > pro-Si306 > Si306 > Si221 (bold values in Table 4). The neutral pro-Si221 had the highest interaction energy −13.75 kcal/mol. Further, pro-Si221 and pro-Si306 had higher scores (better interaction energies) compared to their source structures Si221 and Si306. In our simulation, pro-Si306 showed higher S-values compared to Si306 for both neutral (−12.87 vs. −11.91) and protonated (−13.64 vs. −12.41) forms (Table 4). Thus, the results of the docking are in agreement with the results obtained in both MDR cancer cell lines. For the protonated forms, no definitive trend can be outlined due to the fact that the simulated ionized forms correspond to different percentages of the compounds’ protonation (see Table 3).

To better evaluate the S-scores of the studied compounds, we compared them to the S-scores of TQ (Table 4). The scores of the neutral and protonated TQ were comparable and the lowest among all S-values, suggesting that this inhibitor had a stronger interaction with P-gp compared to the studied compounds.

**Figure 6:**
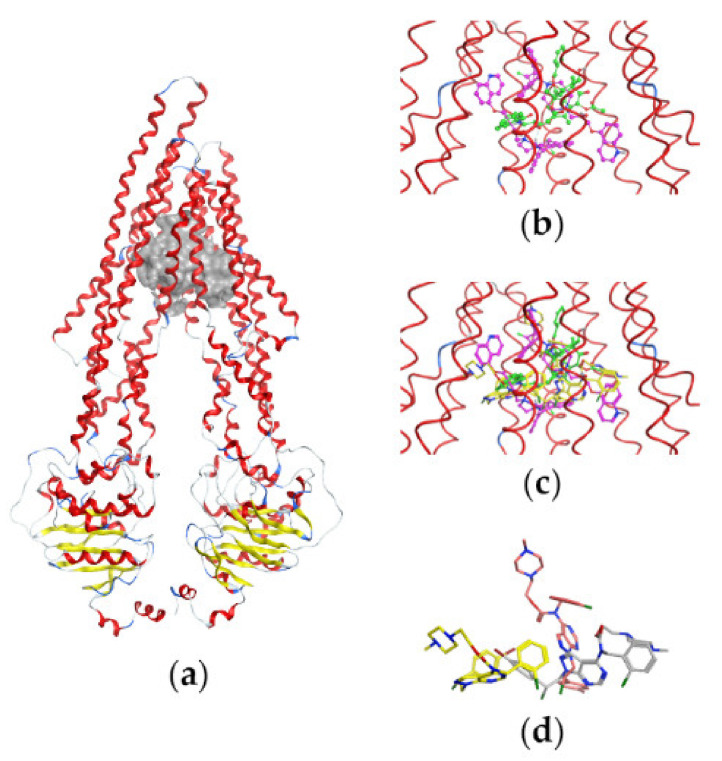
Binding of pro-Si221 in the P-gp cavity. (**a**) A front view of the P-gp structure used in docking studies with the bound substrate PTX (in green) and two molecules of the inhibitor Zosuquidar (in magenta); the protein backbone is shown as a ribbon and is colored according to the secondary structure—loop (in grey), helix (in red), and turn (in blue). (**b**) A closer view of the transmembrane-binding cavity of P-gp with bound PTX and two molecules of Zosuquidar (shown as balls and sticks). (**c**) A closer view of the transmembrane-binding cavity of P-gp with bound PTX, Zosuquidar, and three conformations of pro-Si221 (yellow sticks) corresponding to three different binding poses among the 10 best generated during docking. (**d**) The three different poses of pro-Si221 (shown as yellow, orange, and light gray sticks) positioned equally to those on (**c**). For a better view, the protein backbone is shown as a line ribbon in (**b**,**c**).

Figure 6 illustrates the P-gp structure used in the docking studies with the bound substrate PTX, two molecules of the inhibitor Zosuquidar, and the most active compound pro-Si221 shown in three of its binding poses generated during the simulations. As seen in Figure 6, pro-Si221 can occupy the binding cavity of P-gp simultaneously overlaying the structures of PTX and two molecules of Zosuquidar.

### 2.5. Kinetics of P-gp Inhibition

**Figure 7:**
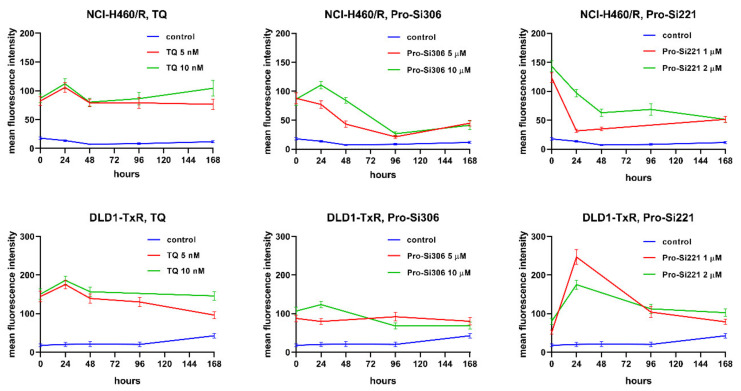
Sustained inhibition of P-gp after acute treatments with pro-Si306 and pro-Si221. Mean fluorescence intensity of Rho 123 accumulation assessed in NCI-H460/R and DLD1-TxR cells treated with pro-Si306 and pro-Si221 for 24 h, 48 h, 96 h, and 168 h. TQ was used as a reference compound. Three independent experiments were performed (a minimum of 10,000 events were collected for each experimental sample).

To investigate whether the suppression of P-gp inhibition could be sustained during a longer period of treatment, the accumulation of Rho123 was analyzed in MDR cancer cells after 24-, 48-, 96-, and 168-h treatments with pro-Si306 and pro-Si221. Their effects on P-gp inhibition were compared with corresponding TQ treatments. The kinetics patterns of 5 nM and 10 nM TQ and of 10 µM pro-Si306 were similar in both MDR cancer cell lines showing a typical increase in P-gp inhibition after 24 h and decrease to the level of starting P-gp inhibition after 48 h (Figure 7). The Pro-Si306 effect continued to decrease and reached stability after 96 h, while TQ reached stable P-gp inhibition after 48 h that was sustained until 168 h (Figure 7). Although pro-Si221 showed the highest starting increase of P-gp inhibition in NCI-H460/R cells, the inhibition declined after 24 h and continued to decrease until 48 h when it reached stability and was sustained even after 168 h (Figure 7). The kinetics pattern of 1 µM and 2 µM pro-Si221 in DLD1-TxR was similar to the pattern of 10 µM pro-Si306, with an increase after 24 h and decline until 96 h when it reached stable P-gp inhibition (Figure 7).

### 2.6. SFK Inhibitors Sensitize MDR Cancer Cells to DOX and PTX

Considering the potential of pro-Si306 and pro-Si221 to suppress P-gp function over time, their ability to reverse DOX and PTX resistance was examined in NCI-H460/R and DLD1-TxR cells, respectively (Table 5). The interactions between SFK inhibitors and DOX as well as between SFK inhibitors and PTX were assessed after 72 h by the MTT assay. Both SFK inhibitors applied at low sub-micromolar concentrations (0.2 µM and 0.5 µM) increased the sensitivity of NCI-H460/R and DLD1-TxR cells to DOX and PTX, respectively. Decreased IC_50_ values for DOX and PTX in all tested combinations are summarized in Table 5. Pro-Si306 showed the highest potential to reverse DOX resistance (10.2-fold) when applied at 0.5 μM (Table 5). The DOX reversal potential of pro-Si221 at 0.2 μM and 0.5 μM increased from 1.8-fold to 7.1-fold, respectively. In addition, pro-Si221 was more efficient in sensitizing DLD1-TxR cells to PTX than pro-Si306 (Table 5).

## 3. Discussion

Src family tyrosine kinases (SFK) are the largest family of non-receptor TKs. Our previous study showed that SFK inhibitors Si306 and its prodrug pro-Si306 suppress the P-gp (ABCB1) transporter activity in MDR glioblastoma cells [21]. Herein, we further evaluated the effect of Si306 and pro-Si306 as well as two additional SFK inhibitors, Si221 and its prodrug pro-Si221, on P-gp efflux pump in MDR non-small cell lung carcinoma and colorectal carcinoma cells. The effect of the tested compounds was compared with a well-known SFK inhibitor, dasatinib. Except for Si221, we showed that the novel SFK inhibitors have a similar or even higher potential to affect cell viability in comparison with dasatinib. Interestingly, SFK inhibitors were more efficient in cells with the MDR phenotype, thus showing collateral sensitivity, a phenomenon in which MDR cancer cells confer higher sensitivity to drugs compared to corresponding sensitive cells [23]. Lower IC_50_ values obtained in MDR cells indicate that analyzed TKIs are not substrates for P-gp. Further, Si306, pro-Si306, and pro-Si221 significantly suppressed P-gp activity. Other authors have also reported that TKIs such as gefitinib [24], osimetrinib [25], and ceritinib [26] exhibited only P-gp-inhibiting activity. Previous studies showed that dasatinib has P-gp inhibitory features if applied at higher concentrations, otherwise it is a P-gp substrate [27]. In our MDR models, 10 µM dasatinib did not show potential to inhibit P-gp. Since it was efficient in inhibiting cell viability at concentrations lower than 10 µM, we assumed that in our MDR models, dasatinib did not have potential to inhibit P-gp. Moreover, its efficacy was lower in MDR non-small cell lung carcinoma cells, thus suggesting that dasatinib is a P-gp substrate.

Our results revealed that Si306, pro-Si306, and pro-Si221 inhibited P-gp activity in a concentration-dependent manner, as evidenced by a progressive increase in the accumulation of Rho123. The highest inhibition of P-gp function was achieved with pro-Si221 owing to the obtained IC_50_ values for P-gp inhibition in a sub-micromolar range. All tested compounds inhibited ATPase activity of P-gp, while the highest effect was exhibited by pro-Si221. Although TKIs usually inhibit ABC transporters by blocking their ATP-binding sites [28,29], our docking simulations showed the binding position of pro-Si221 in the transmembrane cavity of P-gp. Interestingly, it was reported that quinolinone–pyrimidine hybrids also inhibit P-gp activity by binding to its transmembrane domain [30].

The score (S) values obtained from the docking of the studied compounds into the binding cavity of P-gp revealed that ranking of their interaction energy with P-gp (S-ranking) followed the ranking reported for the compounds’ effects on the Rho123 accumulation and inhibition of ATPase activity. The neutral pro-Si221 showed the strongest interaction energy, which is in agreement with its highest effect on ATPase activity and P-gp inhibition in both MDR cancer cell lines. Although prodrugs often have considerably lower biological activity compared to their corresponding drugs [31], it is important to highlight that pro-Si221 and pro-Si306 showed better interaction energies in comparison with their source structures. Docking studies also revealed that the binding site of pro-Si221 overlaps with the binding sites of PTX and Zosuquidar in the transmembrane domain of the P-gp molecule, thus confirming its ability to inhibit the binding of PTX and to block the transport function of the protein, similar to Zosuquidar.

Importantly, both prodrugs showed sustained suppression of P-gp activity up to 168 h. The inhibitory potential of pro-Si221 decreased after 24 h and 48 h in MDR non-small cell lung carcinoma cells but then the effect was sustained over time. In DLD1-TxR cells, the inhibitory pattern was similar to that observed with pro-Si306 in both MDR cancer cell lines, showing an increase after 24 h and a decrease after 48 h until 96 h when the stable suppression of P-gp persisted up to 168 h. According to our evidence, this is the first time that the kinetics of P-gp inhibition were evaluated upon TKI treatments.

TKIs usually inhibit ABC transporters directly and do not affect their expression. However, treatment with TKI imatinib decreased ABCG2 expression, while ponatinib downregulated ABCB1 and ABCG2 cell surface expression [32,33]. Conversely, exposure to gefitinib caused the induction of ABCG2 expression [34]. Similarly, our SFK inhibitors increased P-gp expression in MDR non-small cell lung carcinoma cells. The expression of P-gp is positively regulated by Src downstream of the RAS/RAF/MEK/ERK signaling pathway [35]. As we previously showed that the activity of Src was significantly decreased by Si306 and Pro-Si306 [19], which should lead to the decrease in P-gp expression, the increased P-gp expression in NCI-H460/R cells could be explained only as a compensatory mechanism due to the inhibition of P-gp function. The same effect was observed with TQ in both MDR cancer cell lines. Importantly, our results showed that all tested compounds significantly decreased P-gp expression in MDR colorectal carcinoma cells, implying a cell type differential effect on P-gp expression between non-small cell lung carcinoma and colorectal carcinoma cells.

When TKIs inhibit ABC transporters, drugs that are substrates for these transporters are no longer extruded outside of cells, which increases the cytotoxicity of substrate drugs in resistant cells overexpressing ABC transporters. The administration of various TKIs in combination with anticancer drugs could sensitize MDR cancer cells to paclitaxel an docetaxel [24], vincristine and vinblastine [36,37], doxorubicin [36], and etoposide [38]. Our study showed that sub-micromolar concentrations of prodrugs, significantly lower than those used in the kinetics study of P-gp inhibition, could revert DOX and PTX resistance in MDR cancer cells. This implies that pro-Si306 and pro-Si221 act as P-gp inhibitors regardless of the applied concentration. Importantly, pro-Si306 achieved significant reversal of DOX resistance at 0.5 µM (10.2-fold), while its IC_50_ for P-gp inhibition in NCI-H460/R cells was 6.2 µM.

## 4. Materials and Methods

### 4.1. Drugs

Pyrazolo[3,4-*d*]pyrimidine derivative Si306, its prodrug pro-Si306, as well as Si221 and its prodrug pro-Si221 were obtained as previously described [19]. Dasatinib, PTX, DOX, and Dex-VER were purchased from Sigma–Aldrich Chemie Gmbh, Hamburg, Germany. TQ was obtained from Avant Pharmaceuticals, London, UK. Si306, pro-Si306, Si221, pro-Si221, and dasatinib were dissolved in dimethyl sulfoxide (DMSO) and kept at room temperature as 20 mM aliquots. TQ was also dissolved in DMSO (10 μM stocks) but kept at −20 °C. PTX aliquots were stored at −20 °C in absolute ethanol (1 mM), while 1 mM sterile water dilution of Dex-VER was kept at room temperature. Immediately before treatments, drugs were diluted in sterile deionized water.

### 4.2. Chemicals

RPMI 1640 medium, fetal bovine serum (FBS), antibiotic-antimycotic solution, L-glutamine, and trypsin/EDTA were purchased from Biological Industries, Beit HaEmek, Israel. Rho 123, DMSO and thiazolyl blue tetrazolium bromide (MTT) were obtained from Sigma–Aldrich Chemie GmbH, Hamburg, Germany. FITC-conjugated anti-P-gp antibody was purchased from BD Biosciences, Plymouth, UK. A Pgp-Glo^TM^ assay system was obtained from Promega, Madison, WI, USA.

### 4.3. Cell Culture

NCI-H460 (human non-small cell lung carcinoma) and DLD1 (human colorectal carcinoma) were purchased from the American Type Culture Collection, Rockville, MD. P-gp overexpressing MDR NCI-H460/R cells were selected from NCI-H460 cells after DOX selective pressure [39], while DLD1-TxR cells were selected by continuous exposure to increasing concentrations of PTX from DLD1 cells [40]. All cell lines were grown in RPMI 1640 medium supplemented with 10% FBS, 2 mM L-glutamine, and 10,000 U/mL penicillin, 10 mg/mL streptomycin, and 25 mg/mL amphotericin B solutions. They were sub-cultured at 72-h intervals using 0.25% trypsin/EDTA and seeded in a fresh medium at 8000 cells/cm^2^. Cell lines were maintained at 37 °C in a humidified 5% CO_2_ atmosphere.

### 4.4. MTT Assay

The metabolic activity of viable cells was measured by MTT assay. Cells grown in 25 cm^2^ tissue flasks were trypsinized, seeded into flat-bottomed 96-well tissue culture plates, and incubated overnight. Subsequently, the cells were treated with increasing concentrations of Si306, pro-Si306, and dasatinib (1–25 μM), as well as with Si221 and pro-Si221 (2.5–50 μM) for 72 h. In addition, the combined effects of Si306, pro-Si306, and pro-Si221with DOX as well as with PTX were studied in NCI-H460/R and DLD1-TxR cells, respectively. In simultaneous treatments that lasted 72 h in NCI-H460/R and DLD1-TxR cells, two concentrations of Si306, pro-Si306, and pro-Si221 (0.2 μM and 0.5 μM) were combined with increasing concentrations of DOX and PTX (0.1–2 μM). After treatment, MTT was added to each well at a final concentration of 0.2 mg/mL for 3 h. Then, formazan produced in cells with intact/viable mitochondria was dissolved in 200 μL of DMSO, and the absorbance was measured at 570 nm on a Multiskan Sky Microplate Spectrophotometer (ThermoFisher Scientific, United States). The IC_50_ value is defined as the concentration of a drug that decreases cell viability by 50% and was calculated by nonlinear regression analysis using GraphPad Prism 6.0 for Windows (La Jolla, CA, USA). Relative reversal was calculated as a relation between the IC_50_ values of DOX and PTX alone obtained in NCI-H460/R and DLD1-TxR cells, respectively and the IC_50_ values of DOX and PTX in combination with Pro-Si306 and Pro-Si221. If relative reversal was higher than 2, we considered that tested SFK inhibitors had significant potential to reverse DOX and PTX resistance (SFK inhibitors decreased the IC_50_ for DOX and PTX by more than 2-fold).

### 4.5. Rho 123 Accumulation Assay

The accumulation of fluorescent P-gp substrate Rho 123 was analyzed by flow cytometry. The intensity of fluorescence was proportional to Rho 123 accumulation in the cell. Studies were carried out with Si306, pro-Si306, Si221, pro-Si221, dasatinib, Dex-VER, and TQ in NCI-H460/R and DLD1-TxR cells. NCI-H460 and DLD1 cells were used as a positive control for Rho 123 accumulation. All cell lines were seeded in adherent 6-well plates and grown overnight. In simultaneous treatment that lasted 30 min, 5 μM Rho 123 was applied along with 10 μM of Si306, pro-Si306, Si221, pro-Si221, dasatinib, and Dex-VER. To obtain IC_50_ values for P-gp inhibition, 5 μM Rho 123 was simultaneously applied with increasing concentrations of Si306 and pro-Si306, (1, 2, 5, 10, and 20 μM), pro-Si221, (0.05, 0.1, 0.2, 0.5, 1, 2, 5, 10, and 20 μM) and TQ (1, 2, 5, 10, and 20 nM). Samples were incubated for 30 min at 37 °C in 5% CO_2_. In another experimental setting, the cells were treated with 5 and 10 μM of pro-Si306 and pro-Si221, as well as with 5 and 10 nM of TQ for 24 h, 48 h, 96 h, and 168 h before incubation with 5 μM Rho 123 that lasted an additional 30 min at 37 °C in 5% CO_2_. At the end of the accumulation period, the cells were trypsinized, pelleted by centrifugation, washed with PBS, and placed in ice-cold PBS. The samples were kept on ice in the dark until analysis on a CyFlow Space Partec flow cytometer (Sysmex Partec GmbH, Görlitz, Germany). The fluorescence of Rho 123 was detected on fluorescence channel 1 (FL1-H) at 530 nm. Three independent experiments were performed for each experimental setting. A minimum of 10,000 events was assayed for each sample, and the obtained results were analyzed using Summit Dako Software (ver. 4.3, Fort Collins, CO, USA).

### 4.6. P-gp ATPase Activity Assay

P-gp function depends on ATP hydrolysis energy, and compounds that interact with P-gp can stimulate or inhibit its ATPase activity. P-gp ATPase activity is usually stimulated by its substrates [41]. The Pgp-GloTM assay system detects a compound’s effect on recombinant human P-gp in a cell membrane fraction. P-gp ATPase activity was measured using the luminescent Pgp-Glo^TM^ assay kit according to manufacturer’s instructions. Briefly, ATP was first incubated with P-gp; then, the P-gp ATPase reaction was stopped, and the remaining un-metabolized ATP was detected as a luciferase-generated luminescent signal. P-gp-dependent decreases in luminescence reflect ATP consumption by P-gp; thus, the greater the decrease in signal, the higher the P-gp activity. Accordingly, samples containing compounds that stimulate P-gp ATPase will have significantly lower signals than untreated samples, and vice versa, compounds that inhibit P-gp ATPase will have a higher signal compared to untreated controls. The impact of the tested compounds on P-gp ATPase activity was examined by comparing untreated samples and samples treated with tested compounds to a sodium orthovanadate (Na3VO4)-treated control. Na3VO4 is a noncompetitive inhibitor of P-gp ATPase activity. The difference in the luminescent signal, expressed as relative light units (RLU), between Na3VO4-treated samples and untreated samples represents the basal P-gp ATPase activity (ΔRLU basal). The difference in the luminescent signal between Na3VO4-treated samples and samples treated with the tested compounds represents P-gp ATPase activity in the presence of the tested compounds. Verapamil supplied in the assay kit was used as a positive control as a P-gp substrate and competitive inhibitor. Since verapamil is a P-gp substrate, it caused substantially larger decreases in RLU compared to Na3VO4-treated samples which means that the difference between their luminescent signals (ΔRLU) was significantly higher than ΔRLU basal. The luminescence of samples treated with 5 μM and 20 μM of Si306, pro-Si306, and pro-Si221 was measured using a luminometer microplate reader (CHAMELEONTMV, Hidex, Turku, Finland).

### 4.7. Docking Studies

In silico studies were performed in the software platform MOE v. 2020.09 [MOE, Molecular Operating Environment, Chemical Computing Group Inc., Montreal, QC, Canada].

P-gp 3D structures were downloaded from the Protein Data Bank (PDB) [42] using the following IDs: 6QEX and 6QEE [43]. In the complex 6QEX, the human P-gp has been crystalized with its substrate PTX; in the complex 6QEE, the protein has been resolved with two molecules of its inhibitor Zosuquidar. Both P-gp complexes were initially processed using the “Protonate 3D” tool in MOE at 300K; pH = 7.4, and 0.1 mol/L ion concentration. Both structures were then superposed, and the protein structure from the complex 6QEE was removed. The remaining 6QEX structure with the bound PTX and Zosuqudar structures was then processed by the “Quick preparation” tool in MOE. In addition to adding missing hydrogens and assigning the correct protonation states of the amino acid residues, this tool adds tethers to the receptor heavy atoms and fixes the distant atoms. The system was finally refined using the Amber14: EHT force field with an RMS gradient of 0.1 kcal/mol/Å2, including the specified default tethers. The refined P-gp structure with the ligands was used for docking. The binding site was defined by the ligands PTX and the two Zosuquidar molecules.

The 3D structures of compounds Si221, Si306, pro-Si221, and pro-Si306 were built and optimized in MOE using the force field MMFF94s. The protonation states of the compounds populated at physiological pH = 7.4 were predicted using the “Protomer” tool in MOE and subsequently ordered according to the decreasing percentage of population, %C (Table 3).

The following settings were used in the docking protocol: (i) “Triangle Matcher” placement method; (ii) London dG scoring function (30 placement poses); (iii) refinement with “Rigid receptor” by final scoring using London dG and keeping the 10 best poses.

### 4.8. Flow Cytometric Analysis of P-gp Expression

The P-gp expression level in NCI-H460/R and DLD1-TxR was measured by flow cytometry. MDR cancer cells were seeded in adherent 6-well plates and treated with Si306 and pro-Si306 (5 µM and 10 µM), pro-Si221 (1 µM and 2 µM), and TQ (5 nM and 10 nM). After 48 h, cells were collected by trypsinization, washed in PBS, and then directly immuno stained by FITC-conjugated anti-P-gp antibody according to the manufacturer’s protocol. The samples were kept in the dark until analysis on a CyFlow Space Partec flow cytometer (SysmexPartec GmbH, Germany). The fluorescence of FITC-conjugated anti-P-gp was detected on fluorescence channel 1 (FL1-H) at 530 nm. A minimum of 10,000 events were assayed for each sample, and the obtained results were analyzed using Summit Dako Software (ver. 4.3, Fort Collins, CO, USA).

## 5. Conclusions

The efficacy of many TKIs is limited by the fact that they are substrates for ABC transporters. Therefore, the identification of TKIs whose activity could not be compromised by the overexpression and activity of ABC transporters is an urgent need. Herein, we showed that new SFK inhibitors Si306, its prodrug pro-Si306 as well as pro-Si221, were able to suppress P-gp activity in two resistant cancer cell lines with P-gp overexpression. Hence, three investigated compounds emerged as new promising dual SFK and P-gp inhibitors able to modulate the MDR phenotype. It is noteworthy that both prodrugs (pro-Si306 and pro-Si221) displayed stronger P-gp-modulating potential compared to their parent drugs (Si306 and Si221), which is a rare feature. In addition, the prodrugs showed higher interaction energies in the docking simulations and relevant binding modes in the transmembrane P-gp binding cavity overlapping the binding sites of PTX and Zosuquidar. Importantly, the inhibition of P-gp activity after acute treatment with prodrugs lasted up to 168 h, showing similarity to the TQ kinetics pattern of P-gp inhibition. Pro-Si221 efficiently suppressed the P-gp activity in a sub-micromolar range, which makes this compound particularly interesting for combination studies with anticancer drugs known to be P-gp substrates. All of these characteristics imply that the new SFK inhibitors could be considered a valuable strategy for combating resistant cancers, especially in combination with other chemotherapeutics.

## Figures and Tables

**Table 1:** Decrease in cell viability induced by SFK inhibitors (presented as IC_50_ in µM).

Compounds	NCI-H460	NCI-H460/R	DLD1	DLD1-TxR
Dasatinib	3.931 ± 0.295	6.885 ± 0.605	3.244 ± 0.411	2.900 ± 0.223
Si306	6.235 ± 0.685	1.893 ± 0.184	3.927 ± 0.679	2.564 ± 0.183
Pro-Si306	2.405 ± 0.376	0.897 ± 0.102	2.451 ± 0.346	1.867 ± 0.128
Si221	14.569 ± 1.477	18.136 ± 2.079	34.134 ± 2.322	11.642 ± 1.287
Pro-Si221	4.269 ± 0.509	5.144 ± 0.573	9.798 ± 1.152	5.704 ± 0.543

**Table 2:** P-gp inhibition analyzed by Rho123 accumulation in cells.

IC_50_ (µM) for P-gp Inhibition		
Compounds	NCI-H460/R	DLD1-TxR
TQ ^a^	1.073 ± 0.174	0.925 ± 0.155
Si306	13.267 ± 0.908	6.443 ± 0.667
Pro-Si306	6.167 ± 0.479	2.952 ± 0.300
Pro-Si221	0.612 ± 0.072	0.669 ± 0.061

^a^ IC_50_ ± S.D. values for tariquidar (TQ) are expressed in nM.

**Table 3:** Prevalent forms of the studied compounds at physiological pH = 7.4.

Compounds	Strongest Base pKa ^а^ ± sd ^b^	%C ^c^ at pH = 7.4	Prevalent Form at pH = 7.4 (%)
Si306	6.2 ± 0.1	38	neutral (58%)
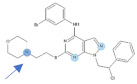			
Pro-Si306	7.5 ± 0.1	61	protonated (99.5%)
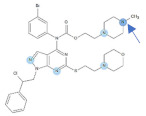			
Si221	3.2 ± 0.1	n.c. ^b^	neutral (98%)
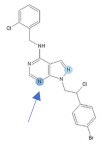			
Pro-Si221	7.5 ± 0.1	96	protonated (99%)
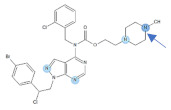			

^a^ pKa values were calculated by ACD/Percepta [ACD/Lab release 2020.2.0, Advanced Chemistry Development, Inc., www.acdlabs.com, accessed on April 2021]; ^b^ sd—standard deviation; ^b^ n.c.—not calculated; ^c^ %C is the concentration of the ionized compound’s form at physiological pH = 7.4 corresponding to the compound’s strongest base as calculated in MOE. The arrow indicates the darkest blue circle in the structures as the atom with the strongest base pKa value (no acid pKa recorded).

**Table 4:** Scores (S) obtained from the docking of the studied compounds into the binding cavity of P-gp.

Compounds	Docking Scores S, kcal/mol			
	Neutral		Protonated ^a^	
	1st Pose	10th Pose	1st Pose	10th Pose
pro-Si221	**−13.75 ^b^**	−10.72	−12.56	−11.13
pro-Si306	**−12.87**	−11.76	−13.64	−11.57
Si306	**−11.91**	−10.17	−12.41	−10.56
Si221	**−11.02**	−9.46	-	-
TQ	−17.28	−14.18	−17.71 ^b^	−14.21

^a^ see Table 3; ^b^ 98% protonated at pH = 7.4; ^b^ The lower S-value indicates a stronger interaction energy: the scores obtained for the neutral and ionized (protonated) forms of highest %C are reported; the compounds are listed in increasing order of the S-values obtained with the neutral forms of the compounds (in bold).

**Table 5:** Relative reversal of DOX and PTX resistance in NCI-H460/R and DLD1-TxR cells induced by SFK inhibitors.

Compounds/Cell Lines	IC50 (nM)	Relative Reversal
DOX/NCI-H460/R	905.4 ± 44.4	
Pro-Si306 0.2 µM	280.7 ± 23.3	3.2
Pro-Si306 0.5 µM	89.0 ± 5.1	10.2
Pro-Si221 0.2 µM	509.3 ± 45.3	1.8
Pro-Si221 0.5 µM	127.2 ± 5.2	7.1
PTX/DLD1-TxR	1052.0 ± 114.5	
Pro-Si306 0.2 µM	707.8 ± 49.9	1.5
Pro-Si306 0.5 µM	418.4 ± 23.6	2.5
Pro-Si221 0.2 µM	537.7 ± 24.4	2.0
Pro-Si221 0.5 µM	331.6 ± 15.7	3.2

## Data Availability

The data presented in this study are available on request from the corresponding author. The data are not publicly available due to lack of an institutional online database.
